# Heterogeneity of rock-hosted microbial communities in a serpentinizing aquifer of the Coast Range Ophiolite

**DOI:** 10.3389/fmicb.2025.1504241

**Published:** 2025-03-07

**Authors:** Katrina I. Twing, William J. Brazelton, Tom M. McCollom, Florence Schubotz, H. Lizethe Pendleton, Rachel L. Harris, Annemarie R. Brown, Seth M. Richins, Michael D. Y. Kubo, Tori M. Hoehler, Dawn Cardace, Matthew O. Schrenk

**Affiliations:** ^1^Department of Microbiology, Genetics, and Immunology, Michigan State University, East Lansing, MI, United States; ^2^School of Biological Sciences, University of Utah, Salt Lake City, UT, United States; ^3^Department of Microbiology, Weber State University, Ogden, UT, United States; ^4^Laboratory for Atmospheric and Space Physics, University of Colorado, Boulder, CO, United States; ^5^MARUM Center for Marine Environmental Sciences, University of Bremen, Bremen, Germany; ^6^Department of Organismic and Evolutionary Biology, Harvard University, Cambridge, MA, United States; ^7^SETI Institute, Mountain View, CA, United States; ^8^Exobiology Branch, NASA Ames Research Center, Moffett Field, CA, United States; ^9^Department of Geosciences, University of Rhode Island, Kingston, RI, United States

**Keywords:** 16S rRNA, serpentinization, subsurface, low biomass, rock hosted

## Abstract

The movement of groundwater through fractured bedrock provides favorable conditions for subsurface microbial life, characterized by constrained flow pathways and distinctive local environmental conditions. In this study, we examined a subsurface microbial ecosystem associated with serpentinized rocks recovered from the Coast Range Ophiolite in northern California, USA. The distribution and diversity of microbial communities at various depths within two separate cores reaching up to 45 m below the land surface were investigated with microbiological and geochemical approaches. Core samples contained low total organic carbon content, low DNA yields, and low copy numbers of *16S rRNA* genes, yet some samples still yielded amplifiable DNA sequences. The microbial community composition of rock cores was distinct from groundwater, and source tracking of DNA sequences indicated that groundwater is not a significant source of DNA into basement rocks. In contrast, the microbial community of some rock core samples shared similarities with overlying soil samples, which could indicate potential contamination, weathering of shallow serpentinites, or a combination of both. Individual DNA sequences of archaea and bacteria predicted to be endemic to the basement rocks were identified by differential abundance analyses. Core-enriched sequences were distinct from those in groundwater or in the overlying soils and included OTUs related to *Serpentinimonas* as well as putatively anaerobic, deep subsurface-associated taxa such as methanogens and *Bathyarchaeia*. Stable isotope analyses of organic and inorganic carbon did not reveal a chemoautotrophic signal and were instead consistent with a primarily surface vegetation source of organic carbon into the basement. This census of archaeal and bacterial DNA sequences associated with altered ultramafic rocks provides a useful resource for further research into the potential for deep subsurface microbial activity fueled by geochemical reactions associated with serpentinization.

## Introduction

The quantity of liquid water held in bedrock at biologically permissive temperatures (<150°C) beneath the oceans and continents is vast, but our understanding of the varied habitats supported in subsurface environments is still being refined. Most insight into rock-hosted subsurface ecosystems has come through analysis of groundwater from drilled wells or surface seeps, while relatively few studies have directly examined microorganisms attached to rock surfaces (Magnabosco et al., 2018; Templeton and Caro, 2023). In contrast to porous sandstones, the flux of water and materials through altered igneous and metamorphic rocks is dominated by preferential flow occurring along fractures and at interfaces between different lithologies. Although these fractures are of particular biological interest, interfaces are often the most difficult to recover by drilling, and identifying small fractures free of contamination within bulk rocks is challenging. One such type of host material, serpentinites, are observed along continental margins as mantle rock is brought to the near surface through tectonic processes. The formation of serpentinites through oxidation and hydration of ultramafic mantle rocks via the process of serpentinization creates high pH (Crespo-Medina et al., 2017; Brazelton et al., 2012; Suzuki et al., 2013; Tiago and Veríssimo, 2013), oxidant-poor fluids rich in reduced gasses (e.g., hydrogen and methane) (Schrenk et al., 2013; Lang and Brazelton, 2020). When exposed at the surface, serpentinite rocks weather into metal-rich, nutrient-poor soils that host endemic flora, as well as distinct microbial communities (Safford and Miller, 2020).

The microbiology of ultrabasic fluids from serpentinite springs (Brazelton et al., 2013; Brazelton et al., 2017; Woycheese et al., 2015; Crespo-Medina et al., 2017; Brazelton et al., 2012; Suzuki et al., 2013; Tiago and Veríssimo, 2013; Trutschel et al., 2022) and boreholes (Twing et al., 2017; Crespo-Medina et al., 2014; Templeton et al., 2021; Fones et al., 2019; Colman et al., 2022) has been previously studied at a variety of locations around the world. These studies have highlighted the ubiquity of a novel genus of alkaliphiles named *Serpentinimonas* in high pH serpentinizing fluids (Suzuki et al., 2014; Bird et al., 2021). The presence of methanogens, sulfate-reducing bacteria, and fermentative bacteria apparently fueled by the abundance of dihydrogen and low-molecular-weight organic compounds also seems to be a common theme in these fluids (Templeton and Caro, 2023; Schrenk et al., 2013; Templeton et al., 2021). However, microbiological investigations of serpentinite bedrock have proven to be more challenging due to the even lower cell densities per unit volume in rock samples than in fluids and the technical difficulties of isolating and purifying DNA from serpentinized rocks. Shallow cores into seafloor serpentinites revealed a variety of potentially novel, uncultivated archaea and bacteria that did not include any taxa previously identified in continental serpentinite springs or borehole fluids (Motamedi et al., 2020; Goordial et al., 2021). To our knowledge, only one previous census of bacteria and archaea within competent serpentinized bedrock in a continental setting has been reported (Kraus, 2021).

In this study, we report a census of microbial diversity in rock cores from two boreholes (31–45 m deep) in the Coast Range Ophiolite of northern California, along with associated geochemical and geophysical data. We have previously reported on the microbial ecology and geochemistry of fluids from these boreholes, which were characterized by low oxygen (DO 0.03–0.05 mg/L) and extreme high pH (pH 11.5–12.2) and dominated by *Serpentinomonas* and *Clostridia*. Here, our results of the rock core samples include the unexpected discovery of *Bathyarchaeia*, as well as methanogens, in the drill cores and highlight the connectivity among serpentinized bedrock, ultrabasic groundwater, and serpentine soils.

## Materials and methods

### Site description and sample collection

The Coast Range Ophiolite Microbial Observatory (CROMO) was established in August 2011, when eight wells were drilled into an aquifer hosted in heavily serpentinized peridotite at the UC Davis Donald and Sylvia McLaughlin Natural Reserve near Lower Lake, California. Details of the drilling operations can be found in Cardace et al. (2013). Briefly, two main wells, CSW1.1 and QV1.1, were drilled 1.4 km apart to depths of 31 m and 45 m, respectively, using HQ wireline coring with an inner diameter of 63.5 mm. To mitigate contamination during drilling, purified water (filtered through a 0.1 μm filter and ozonated) was used as the drilling fluid, and 0.5 μm fluorescent microbead tracers (Polysciences Inc.) were included in the drill stream at a concentration of 10^4^ beads mL^−1^ (Cardace et al., 2013). Samples for contaminant detection were collected on site from separate interior and exterior sections of whole round cores, preserved in 4% paraformaldehyde, and examined by epifluorescence microscopy. Only samples free of visible fluorescent beads (~85% of the total core samples) were used in downstream microbiological analyses. Fluorescent beads were primarily evident in regions of unconsolidated material, rather than solid rock. Cores were cataloged, and sub-samples were preserved for complimentary mineralogical, geochemical, and microbiological analyses. Microbiological samples were wrapped in combusted aluminum foil, placed into sterile Whirlpak bags, and frozen with liquid nitrogen on-site and subsequently stored at −80°C until DNA extraction.

Additionally, soil samples were collected from the area adjacent to the wells in December 2013 using a sterile micro-coring approach, resulting in approximately 13-cm-long soil cores. Plant and root materials were removed from the cores before they were divided lengthwise to create the replicate soil samples (QV_soil1/QV_soil2 and CSW_soil1/CSW_soil2, respectively) and flash-frozen in the field laboratory. The soil samples were stored frozen until processing with the same DNA extraction methods used for the rock core samples. Groundwater samples from the CSW1.1 and QV1.1 wells were collected in August 2013 by filtration through 0.2 μm Sterivex cartridges and processed following analyses previously described by Twing et al. (2017). During another sampling campaign in 2014, the potential presence of cells and viruses in the 0.2 μm filtrate was explored by concentration of >10 L of filtrate with a VivaFlow 200 concentration cassette (Hydrosart 30k MWCO). The DNA yield of these concentrated filtrates was below detection, and amplicon sequencing of the bacterial *16S rRNA* gene was not successful.

### Mineralogical and geochemical analyses

The minerals in the core samples were characterized using a combination of X-ray diffraction (XRD) and scanning electron microscopy coupled with energy dispersive X-ray spectroscopy (SEM/EDS). Analyses by XRD were used to identify major mineral components of the cores (>5%) and were performed on powdered bulk core samples using a Terra instrument (Olympus, Inc.) with Co Kα radiation. Additional characterization of the minerals, as well as identification of minor components, was performed using SEM/EDS analysis. The analyses were performed using a Hitachi SU3500 SEM equipped with an Oxford Instruments EDS and AZTEC data processing software. Observations were made on crushed core pieces mounted on Al stubs using carbon tape.

### Total carbon and total organic carbon analyses

Total carbon (TC) and total organic carbon (TOC) content and stable carbon isotopic composition (δ^13^C) was measured with a Thermo Scientific Flash 2000 elemental analyzer coupled to a Thermo Delta V Plus isotope ratio mass spectrometer. TOC content and δ^13^C was determined following decalcification. For this, ground and homogenized samples were treated with hydrochloric acid (12.5%, aq.), neutralized with deionized water and subsequently freeze-dried. TC and TOC were quantified using a laboratory standard of estuarine sediment and accounting for the weight loss during decalcification. Measured δ^13^C values were calibrated with reference CO_2_ gas (Air Liquide, 99.99% CO_2_). The precision of δ^13^C is better than ±0.39‰, and the accuracy is better than ±0.27‰ based on repeated measurements. For analyses with carbon contents <10 μg, the accuracy decreases to ±2‰. Samples with such low carbon contents are reported in Supplementary Table S1 in parentheses. The δ^13^C values are expressed relative to VPDB (Vienna Pee Dee Belemnite).

### DNA extraction

Thawed core samples and soil material were homogenized using autoclaved and ethanol-sterilized steel percussion mortars and ceramic mortars and pestles. DNA was extracted from two parallel samples of 10 g homogenized core using the PowerMaxSoil Kit (MoBio, Carlsbad, CA, United States), following the manufacturer’s instructions. The resulting DNA suspensions were pooled from replicate extractions and concentrated in an Amicon Ultra-2 Centrifugal Filter Unit with Ultracel-30 membrane (Millipore, Darmstadt, Germany) to a volume of 50 μL. DNA was quantified using High Sensitivity reagents for a Qubit^®^ 2.0 Fluorometer (Life Technologies, Grand Island, NY, United States). To conserve DNA for downstream analyses, only 2 μL of the total 50 μL DNA extract was designated for DNA quantification, providing a detection limit of 0.1 ng/μL of the fluorometric quantification method. Since DNA was extracted from approximately 20 g of core material per sample, the limit of detection for DNA quantification was ~0.25 ng of DNA per g of core material.

### Quantitative-PCR

The abundances of bacteria and archaea in the DNA extracts were determined by quantitative polymerase chain reaction (q-PCR) using domain-specific primers targeting the V6 hypervariable region of the *16S rRNA* gene for archaea and bacteria, as previously described (Méhay et al., 2013). Primer details can be found in Supplementary Table S2. Samples were analyzed on a BioRad C-1000 Thermo-Cycler with a q-PCR module using the SsoAdvanced SybrGreen Assay (Bio Rad, Hercules, CA, United States). Gene copy numbers were calculated using standard curves generated by amplification of DNA from *Methanocaldococcus jannaschii* for archaea and *Escherichia coli* for bacteria. Amplification efficiencies were 96% for the archaeal and 108% for the bacterial qPCR reactions from cores and soils.

### *16S rRNA* gene sequencing and data analysis

Purified DNA samples from core, soils, and groundwater were submitted to the Josephine Bay Paul Center at the Marine Biological Laboratory (MBL) for sequencing of the V4–V5 region of the *16S rRNA* gene on an Illumina MiSeq instrument as part of the Census of Deep Life project (Morrision et al., 2013) using domain-specific primers to target bacteria and archaea, respectively (Supplementary Table S2). New sequence data from rock cores, soils, and groundwater described here are available via BioProject PRJNA1097798. The paired-end reads were merged and subjected to MBL’s post-processing quality control for removal of low-quality reads and chimera checking (Huse et al., 2014). The samples yielded 8,493 to 246,345 and 12,020 to 241,730 merged, quality-filtered sequences with the bacterial and archaeal primers, respectively. Any sample that produced less than 5,000 sequences was considered a failed sequencing attempt and was not included in subsequent analyses.

Amplicon reads were dereplicated into unique sequences using Mothur (Schloss and Westcott, 2011), formed into operational taxonomic units (OTUs) at the 97% sequence similarity level using the Opticlust parameters in Mothur (Westcott and Schloss, 2017), and assigned taxonomy by alignment to the SILVA database (v138; Pruesse et al., 2007; Quast et al., 2013). One thousand four hundred and thirty three OTUs belonging to the genera outlined by Sheik et al. (2018) as putative kit contaminants were removed from the dataset, a detailed list of which can be found in Supplementary Table S3. Additionally, any sequences classified to domain “NA” or “Eukaryota” and all bacteria classified as “Chloroplast” or “Mitochondria” were removed from the dataset. Rarefaction curves were computed in Mothur v1.39.5 (Schloss, 2020; Schloss et al., 2009). Beta diversity was assessed in Mothur using the tree.shared command with the Sørensen dissimilarity index and with an MDS plot with the Bray–Curtis dissimilarity index using the R package Phyloseq v1.26.1 and the plot_ordination command (McMurdie and Holmes, 2013).

SourceTracker2 (Knights et al., 2011) was used to estimate the proportion of the microbial community of each core sample that could be attributed to groundwater or soil. This approach was used to identify samples that contained significant numbers of OTUs associated with groundwater or soil and to remove them from downstream differential abundance analyses. Significant differences in the abundances of OTUs between groups of samples were tested with the aid of the Phyloseq and EdgeR (Robinson et al., 2010) packages in R, which allowed the identification of individual OTUs that were statistically more “enriched” in a given type of sample (i.e., core, soil, or groundwater). OTUs that were significantly more abundant in core samples than in soil or fluid samples were identified as “core-enriched.” Representative sequences of core-enriched OTUs comprising 1% or more of total sequences in at least one sample were searched against the NCBI non-redundant database using BLAST (Altschul et al., 1997) to identify the environmental source of the best hit.

### Phylogenetic analysis of core-enriched *Bathyarchaeia*

A total of 102 “core-enriched” OTUs classified as members of the archaeal phylum *Candidatus* “Bathyarchaeota” (currently recognized in the SILVA database as class *Bathyarchaeia* within phylum Thermoproteota) were aligned with Clustal Omega v.1.2.4 (Sievers and Higgins, 2014) against 498 sequences belonging to 25 Ca. “Bathyarchaeota” subgroups and three outgroups (*Crenarchaeota*, Ca. “Korcharchaeota,” and Ca. “YNPFFA”), concatenated in a previous review by Zhou et al. (2018) and appended with additional sequences from Harris (2020) and Harris et al. (2018). Gaps were removed from aligned sequences using Jalview v.2.11.0 (Waterhouse et al., 2009), and a maximum likelihood tree was inferred via IQ-TREE v.1.6.12 (Nguyen et al., 2015) using UFBoot2 (Hoang et al., 2018) for 1,000 bootstrapping iterations of a TIM3e + G4 model determined from ModelFinder (Kalyaanamoorthy et al., 2017).

### Statistical analyses

The ANOSIM test using a Bray–Curtis dissimilarity index was used to evaluate whether individual environmental parameters had significant effects on the community composition of core samples using the R package vegan (Oksanen et al., 2020). To statistically determine which combinations of numerical environmental variables best explained the community composition variation within the dataset, the bioenv analysis (Clarke, 1993) was performed.

## Results and discussion

### Lithological and geochemical changes along the core depth profile

**Figure 1:**
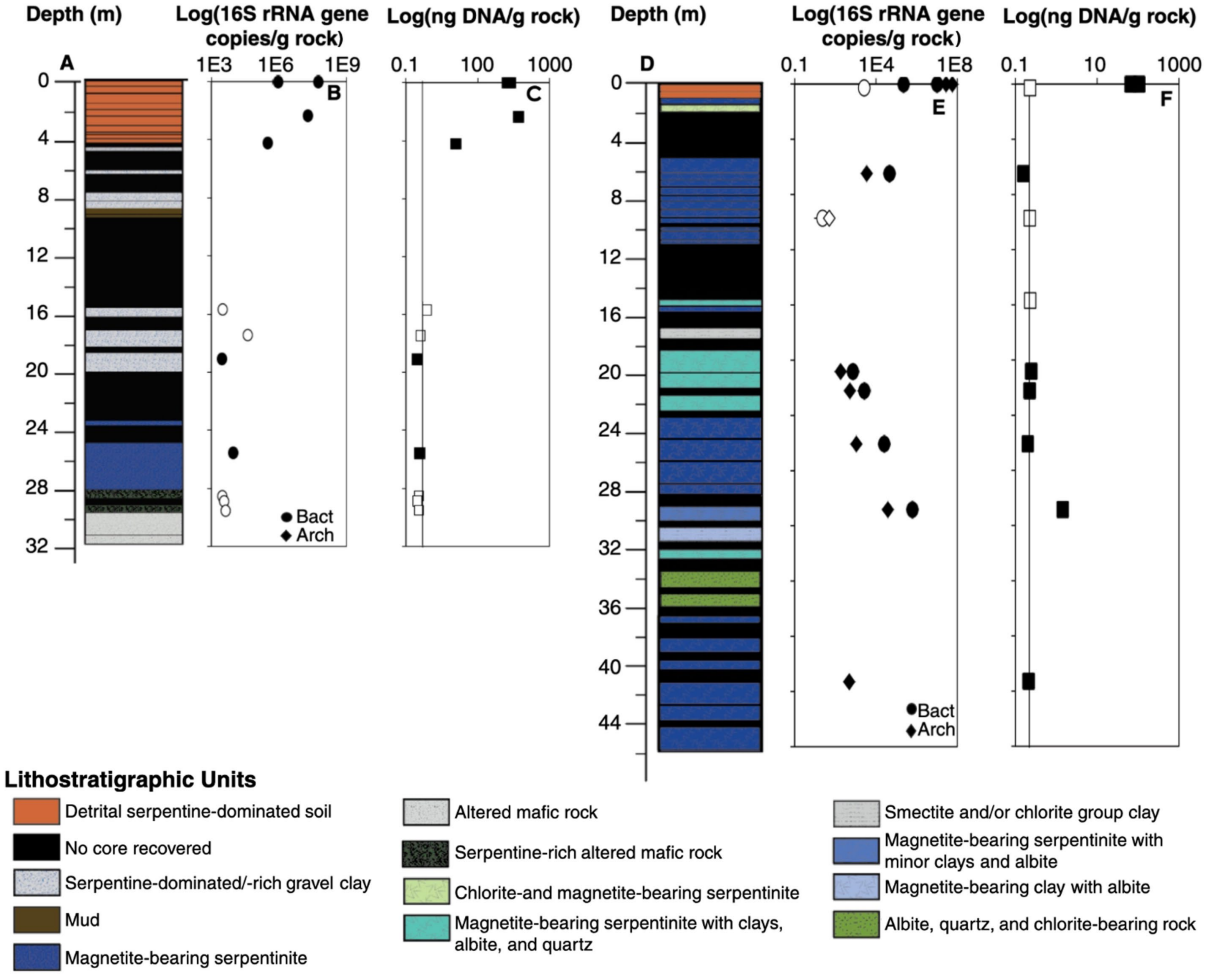
Distribution of microbial biomarkers relative to lithostratigraphic variations. Total genomic DNA was extracted and quantified from subsamples from different lithostratigraphic regions of the CROMO cores at both the CSW **(A)** and QV **(D)** sites. Quantitative-PCR was used to measure the abundance of archaeal (diamonds) and bacterial (circles) amplicons across the samples **(B,E)**. DNA yield was quantified by fluorometric methods **(C,F)**. Filled symbols represent samples that yielded *16S rRNA* gene sequences, samples represented by closed symbols failed sequencing. This figure has been adapted from Cardace et al. (2013), licensed under CC BY 3.0 and Ortiz et al. (2018) with permission from John Wiley & Sons.

Geochemical and mineralogical analyses of the CROMO cores showed minor lithological variations between the surface and total depth of the borehole (Figure 1). The upper few meters of both the QV and CSW sites were represented by unconsolidated serpentine soils, with a gradation into serpentinized bedrock with varying amounts of magnetite and other accessory minerals at depth. The amount of clay minerals, and therefore the permeability of rock samples, varied significantly along the core, reinforcing the interpretation that closely spaced wells are relatively hydrologically isolated at CROMO both laterally and vertically (Ortiz et al., 2018). In addition, relatively silica-rich layers at certain depths of the core (Table 1), particularly at the QV site, may represent intercalary layers that could be coincident with or post-date ophiolite emplacement. These observations are consistent with the extensive ore mining in the region that also reflects hydrothermal processes (Peters, 1993).

**Table 1:** Mineralogical and microbiological data for each core, soil, and fluid sample.

Sample	Depth (mbs)	Mineralogy	DNA yield (ng/g)	Bacterial *16S rRNA* gene			Archaeal *16S rRNA* gene		
				qPCR (copies/g)	qPCR Std. Dev.	Sequence reads^a^	qPCR (copies/g)	qPCR Std. Dev.	Sequence reads^a^
CSW1.1A	31.1	Fluids	5.29	**—**	**—**	**31,054**	—	—	0
CSW1.1B	31.1		2.92	**—**	**—**	**23,413**	—	—	0
CSW1.1C	31.1		5.18	**—**	**—**	**28,051**	—	—	0
CSWsoil1	0	Serpentine soil	67.0	5.73 × 10^7^	2.07 × 10^7^	**42,570**	2.87 × 10^7^	2.10 × 10^6^	**151,538**
CSWsoil2	0		82.1	9.09 × 10^5^	2.64 × 10^5^	**82,363**	3.00 × 10^8^	3.64 × 10^7^	**236,134**
CSW1R	2.4	Mostly quartz, minor albite, unknown phyllosilicate, trace serpentine minerals	138.1	1.96 × 10^7^	1.82 × 10^6^	**246,412**	ND	ND	**89,017**
CSW2R	4.2	Serpentine, quartz, minor albite, unknown phyllosilicate	2.4	3.28 × 10^5^	1.11 × 10^5^	**147,623**	ND	ND	**105,289**
CSW16R	15.7	Serpentine, minor unknown phyllosilicate	0.4	3.20 × 10^3^	1.91 × 10^2^	18	ND	ND	0
CSW17R	17.4	Chlorite, saponite, unknown minerals	0.3	4.17 × 10^4^	4.13 × 10^3^	16	ND	ND	0
CSW10R	19.1	Unknown phyllosilicate, chlorite	<0.2	2.95 × 10^3^	5.71 × 10^2^	**185,703**	ND	ND	**12,020**
CSW14R	25.6	Pure serpentine minerals	<0.2	9.40 × 10^3^	7.87 × 10^2^	**225,988**	ND	ND	0
CSW22R	28.5	Serpentine, saponite, minor actinolite, talc	<0.2	3.08 × 10^3^	5.81 × 10^2^	9	ND	ND	0
CSW23R	28.9	Serpentine, minor talc, saponite	<0.2	3.70 × 10^3^	2.93 × 10^2^	9	ND	ND	0
CSW24R	29.5	Chlorite, actinolite, minor saponite, unknown minerals	<0.2	4.33 × 10^3^	8.30 × 10^2^	11	ND	ND	0
QV1.1A	45.7	Fluids	13.7	**—**	**—**	**36,790**	—	—	0
QV1.1B	45.7		7.31	**—**	**—**	**35,185**	—	—	0
QV1.1C	45.7		19.5	**—**	**—**	**38,145**	—	—	0
QVsoil1	0	Serpentine soil	66.4	2.27 × 10^5^	3.52 × 10^4^	**44,333**	2.81 × 10^7^	3.72 × 10^6^	**193,560**
QVsoil2	0		106.0	1.02 × 10^7^	4.05 × 10^6^	**83,590**	5.69 × 10^7^	2.50 × 10^6^	**242,101**
QV3R	0.3	Pure serpentine minerals	<0.2	2.62 × 10^3^	8.31 × 10^2^	1,919	ND	ND	2,806
QV7R	6.5	Pure serpentine minerals	0.2	4.67 × 10^4^	3.63 × 10^3^	**78,108**	3.52 × 10^3^	4.63 × 10^2^	4,683
QV11R	9.7	Pure serpentine minerals	<0.2	2.37 × 10^1^	1.45 × 10^1^	4,635	5.04 × 10^1^	2.85 × 10^0^	266
QV13R	15.7	Pure serpentine minerals	<0.2	ND	ND	4,568	ND	ND	1,440
QV18R	20.8	Mostly saponite, trace clinochlore, no serpentine	<0.2	7.30 × 10^2^	2.03 × 10^2^	**9,225**	1.84 × 10^2^	4.22 × 10^1^	504
QV21R	22.2	Nearly pure serpentine, minor unknown minerals	<0.2	2.68 × 10^3^	1.45 × 10^3^	**10,827**	5.17 × 10^2^	3.75 × 10^1^	998
QV25R	26.1	Mostly saponite, trace clinochlore, no serpentine	<0.2	2.55 × 10^4^	1.01 × 10^3^	**8,495**	1.09 × 10^3^	2.62 × 10^2^	2,486
QV30R	30.8	Quartz, albite, trace unknown phyllosilicates	1.4	6.21 × 10^5^	3.58 × 10^4^	**25,322**	3.85 × 10^4^	2.38 × 10^3^	**103,561**
QV42R	43.3	Pure serpentine minerals	<0.2	ND	ND	**11,414**	4.85 × 10^2^	2.04 × 10^1^	**130,842**

ND, not detected; —indicates that sample was not tested.

a Sequence counts in bold were successfully sequenced, while those in regular font failed sequencing (i.e., produced <5,000 sequence reads).

Subsamples of the core material were used for EA-irMS analyses to quantify the total carbon (TC) and total organic carbon (TOC) in solid phases, and to determine their stable carbon isotope compositions. TOC contents decrease with depth, going from 3–7 wt% in the soils to values approaching detection limits in deep sections of the core (Supplementary Table S1). Interestingly, a region relatively enriched in TOC (0.03–0.16 wt%) was observed overlying the silica rich samples of the QV core at approximately 21–26 m depth below the surface. The δ^13^C of TOC of the cored section is on average − 25.3‰ ± 0.7‰, and compares to the soil δ^13^C (−25.5‰ ± 0.8‰). While TC contents are also low, ranging from 0.04 to 1 wt%, the samples are overall dominated by inorganic carbon phases, which is reflected in the comparably enriched δ^13^C-TC values ranging from −21.6 to −7.3‰. The ^13^C-depletion of TOC relative to the calculated inorganic carbon δ^13^C values (Ca. −21‰ to −3‰) does not fall into the typical range of isotopic fractionations involved during autotrophy, apart from some methanogens using the reductive acetyl CoA pathway (House et al., 2003). Instead, the consistent isotope values of organic carbon similar to vegetation-associated TOC points to mainly residual surface material encrusted in the rock matrix.

### Quantification of DNA yield and *16S rRNA* genes

Environmental DNA yields from most of the rock core samples were below the detection limit of 0.25 ng of DNA per g of core material (Table 1). The exceptions include the shallowest core samples (2–17 m depth) from CSW and two core samples from QV that contain mostly soil-derived OTUs (see below). These results indicate very low biomass in the serpentinite basement rock compared with nearby serpentine soil and groundwater, which is consistent with the TOC measurements reported above. Similarly, bacterial and archaeal *16S rRNA* genes were much more abundant in the top few meters of the cores, as estimated by quantitative PCR, decreasing from 10^7^–10^8^ gene copies per gram of core in the surface, respectively, down to approximately 10^3^ gene copies per gram in core samples below 20 m depth (Figures 1B,E and Table 1). One sample (QV30) at 30.8 m depth had 10^5^ gene copies per gram; this sample contained mostly soil-affiliated OTUs, as discussed below. A shallow sample (QV11 at 9.7 m depth) had a low value of 10 gene copies per gram, which we consider to be an outlier. Several samples, including all archaeal *16S rRNA* qPCR reactions from CSW core samples, yielded no detectable signal. In general, bacterial *16S rRNA* genes were more abundant than archaeal *16S rRNA* genes in samples where both were detected (Figures 1B,E and Table 1).

Despite unquantifiable DNA concentrations, many of the core samples were amplifiable via domain-specific qPCR and successfully yielded *16S rRNA* gene sequences (Table 1). Therefore, amplifiability, as opposed to DNA concentration, was used to determine whether a sample was fit for submission to the sequencing facility. Low levels of input DNA for amplicon sequencing are known to cause biases in alpha and beta diversity statistics (e.g., Multinu et al., 2018), but such PCR biases among species are unlikely to alter the general conclusions of this initial, exploration-oriented study. For example, the exact relative abundance of a given individual species in low-biomass rocks should be treated with caution, but its presence in the rocks can be nevertheless validated with the contamination-tracing procedures described here. Due to the potential for contamination in very low biomass samples, blank control samples were run at every step of the DNA extraction and purification process. The control samples were neither quantifiable by fluorometric methods nor amplifiable via domain-specific qPCR; therefore, they were not sequenced.

### Bacterial compositions of rock core, groundwater, and soil

Bacterial *16S rRNA* gene amplicon sequences from all rock core, groundwater, and soil samples were clustered into operational taxonomic units (OTUs) and ranged from 2,618–14,160 OTUs in core samples, 165–200 OTUs in groundwater samples, and 4,085–7,615 OTUs in soil samples. The number of OTUs in core and soil samples were roughly correlated with sequencing yield (Supplementary Figure S1), but much lower in groundwater samples even at comparable sequencing depths, consistent with our previous study (Twing et al., 2017).

The overall bacterial community compositions of core, groundwater, and soil samples were distinct (Supplementary Figure S1); i.e., the bacterial communities of core samples from different boreholes (CSW and QV, which are 1.4 km apart) are more similar to each other than to groundwater and soil samples from the same borehole, with two exceptions (QV7R and QV30R) discussed below. CSW cores contained more Chloroflexi and *Gammaproteobacteria* and fewer Actinobacteriota sequences compared to the soil samples and QV cores (Supplementary Figure S2). The presence of Acidobacteria, Actinobacteria, Bacteroidota, Chloroflexi, Planctomycetes, Proteobacteria and Verrucomicrobia in serpentine soil is consistent with previous studies (Supplementary Table S4). Groundwater samples were dominated by Comamonadaceae (previously of the Betaproteobacteria, classified within class *Gammaproteobacteria* in SILVA v138) and Firmicutes, as reported previously (Twing et al., 2017).

### Archaeal compositions of rock core, groundwater, and soil

Numbers of archaeal OTUs in rock core and soil samples ranged from 1,238–24,985 OTUs and correlated with sequencing depth (Supplementary Figure S3). No archaeal sequences were recovered from groundwater samples in this study or by Twing et al. (2017) (Table 1). Archaeal DNA sequences identified in serpentine soil samples consisted exclusively of *Nitrososphaeria* (Supplementary Figure S4), which have been identified among additional archaeal taxa in another report of serpentine soils (Solano-Arguedas et al., 2022) (Supplementary Table S4). In contrast, core samples collected from both boreholes contained a remarkable diversity of archaeal DNA sequences, including *Bathyarchaeia*, *Methanococci*, *Methanosarcinia*, and ANME-1. Core sample QV30R, however, consisted almost entirely of *Nitrososphaeria*, suggesting a strong similarity to overlying soil communities.

### Source-tracking of core microbes to soil and groundwater

We employed a source tracking approach to identify archaeal and bacterial DNA sequences that were most likely to have originated in the rock cores, rather than in the soil or groundwater. It is expected that the basement rock, soil, and groundwater in this system are in communication with each other over geologically short time scales (consistent with the isotope signatures of TOC reported here), so we expect the rock core samples to contain some contribution of microbes derived from soil or groundwater. Therefore, we applied multiple statistical methods to identify sequences that were significantly more abundant in a particular sample type (i.e., core, soils, or groundwater), and therefore, from where they were most likely to have originated.

First, SourceTracker2 (Knights et al., 2011) was used to identify any core samples that have a majority of OTUs derived from groundwater or soil, since such samples would confuse the downstream identification of core-enriched sequences. The “source” samples were grouped: all four soil samples were grouped as the source “soil,” and all s groundwater samples were grouped as the source “fluid.” Each core sample was assessed individually to determine what percentage of the core sample OTUs could be attributed to one of the given sources. The percentage of OTUs identified as “unknown” represents sequences that could not be attributed to one of our known sources (i.e., soil or groundwater) and are therefore potentially endemic to the rock core.

**Figure 2:**
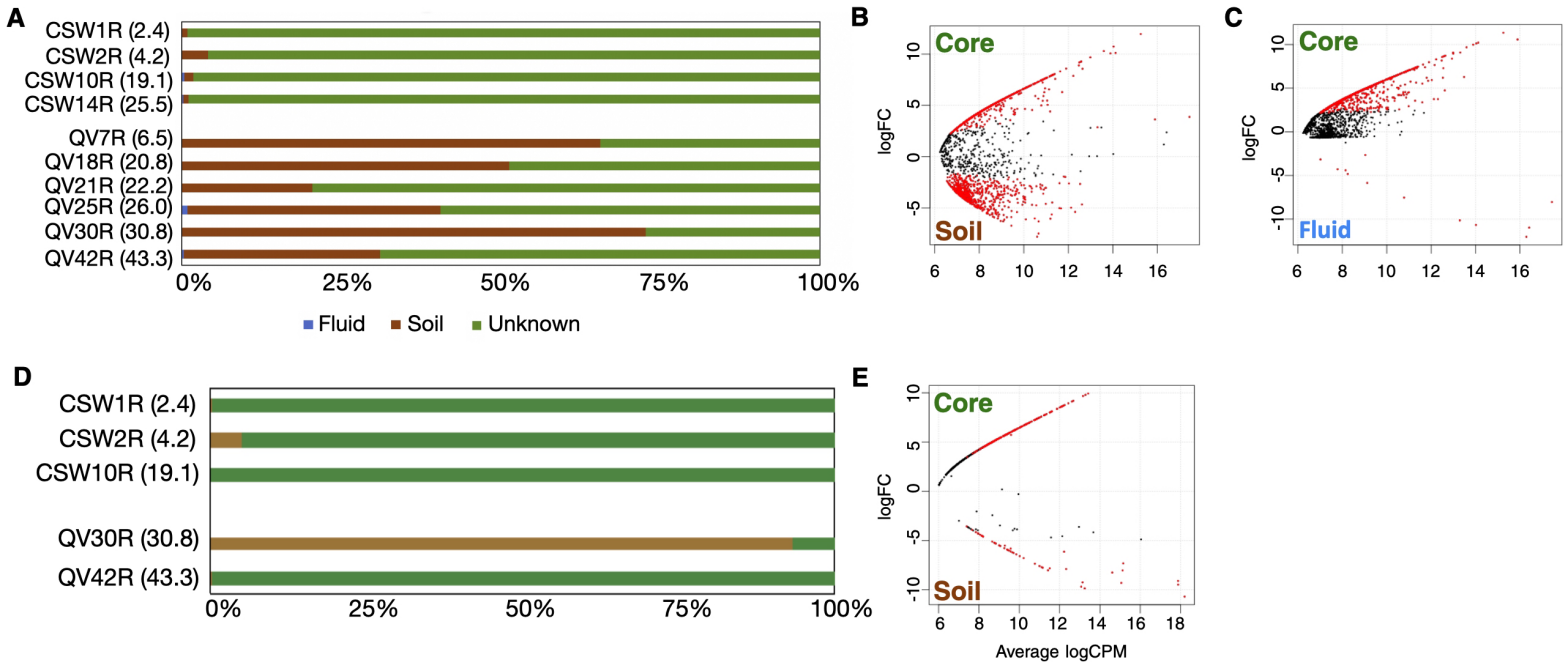
Statistical methods were used to identify endemic core microbes. SourceTracker2 was used to identify the percent of each core community that likely came from either soils (brown) or fluids (blue) for bacteria **(A)** and archaea **(D)**. Samples with greater than 50% contribution of OTUs from soils and fluids (QV7R, QV18R, and QV30R for bacteria; QV30R for archaea) were removed from the “core” classification for downstream differential expression analysis in EdgeR, used to identify core-enriched sequences (red). Comparisons were performed for bacterial data between core and soil samples **(B)** and core and fluid samples **(C)** and archaea between core and soil samples **(E)**.

**Figure 3:**
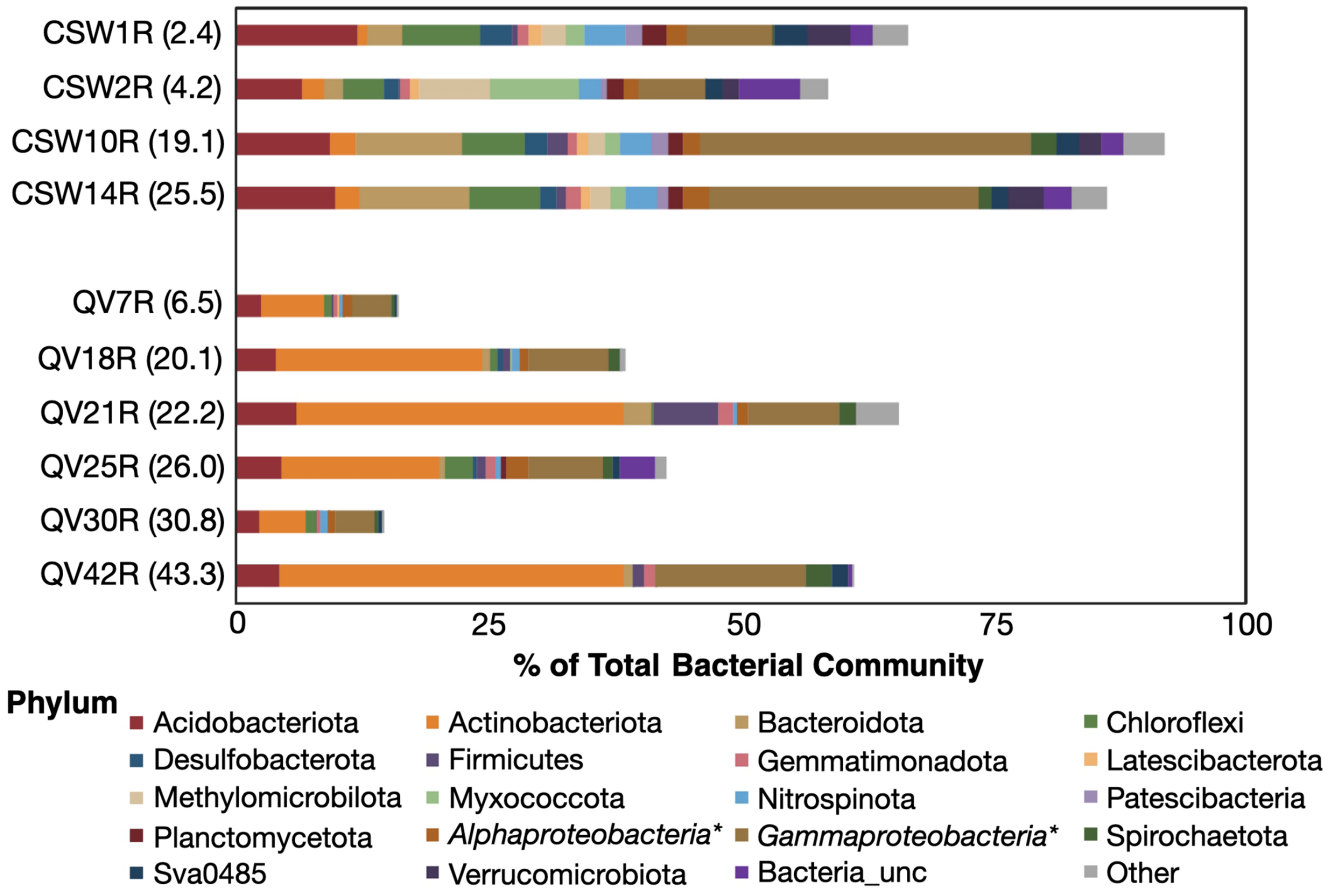
Relative abundance of core-enriched bacterial taxa. The sizes of the bars represent the percent of the bacterial community composed of core-enriched bacterial taxa, with white space beyond the bar representing the portion of the microbial community that could not be attributed to core-enriched taxa (as defined by the differential abundance metrics). The core-enriched taxa are identified at the phylum level, except for the Proteobacteria, which are represented at the class level (*Alphaproteobacteria* and *Gammaproteobacteria*).

Very few core OTUs were traced to groundwater samples (Figure 2), indicating that groundwater is not a significant source of OTUs into the rock core samples. This finding is consistent with a similar study where microbial DNA source tracking indicated a surprisingly small contribution of seawater-derived microbes into seafloor serpentinite rock core samples (Motamedi et al., 2020). In contrast, many rock core OTUs were traced to soil samples, particularly with regard to the bacterial sequences from QV cores (Figure 2A). Dispersal of microbes from soil into basement rocks could occur naturally in the environment or during field sampling or laboratory handling of these low-biomass samples. In the case of the shallowest core samples, it is likely this community overlap represents a transition between the soil layers and basement rock. Soil-associated OTUs in deeper core samples could reflect weathering of basement rock into soil, or potential contamination of deeper core samples with soil material. In order to identify archaea and bacteria that are endemic to relatively unweathered basement rock, core samples with greater than 50% of OTUs traced to soil (i.e., QV7R and QV30R for bacteria; QV30R for archaea) were removed from the “core” category for downstream differential expression analyses (Figures 3A,D). The similarity of these samples to soils is further supported by beta-diversity analyses, in which QV7R and QV30R cluster with soil samples rather than with other core samples (Supplementary Figures S1, S3). Although these samples were not included in the pooled core data for the differential abundance analyses, the relative abundances of “core-enriched” OTUs are still reported for these samples in tables and figures.

### Differential abundance

We used a differential abundance approach to identify specific OTUs that are significantly enriched in rock cores and therefore likely to be endemic to unweathered basement rock. This approach was chosen instead of a “simple overlap” approach, in which all OTUs that occur in both a control sample and a sample of interest are identified as contaminants (e.g., Salter et al., 2014), because such an approach would not be suitable for the present study, where we expect some degree of environmental mixing between the habitats (Motamedi et al., 2020). Instead, we identified OTUs that were significantly enriched in core samples (called “core-enriched”) by comparing the distributions of OTU relative abundances in pooled core samples with their relative abundances in pooled groundwater and soil samples with a differential abundance approach (McMurdie and Holmes, 2013; Robinson et al., 2010). A total of 1,087 bacterial OTUs and 282 archaeal OTUs were identified as core-enriched (Figure 2 and Supplementary Table S5). Of those, 48 bacterial and 36 archaeal OTUs had a relative abundance of ≥1% in at least one core sample (Supplementary Table S6).

### Core-enriched bacteria

A total of 1,087 bacterial core-enriched OTUs were identified across the dataset (Supplementary Table S5), but only 48 of those were >1% relative abundance in any given core sample (Supplementary Table S6). The bacterial OTUs identified as core-enriched in each core sample comprised 14–92% of the total bacterial community (Figure 3). Those on the lower end were QV7R and QV30R, which contain mostly soil-derived OTUs (Figure 2A). The majority of OTUs in samples from the CSW core were identified as core-enriched; therefore, the overall taxonomic distribution of OTUs in these samples did not differ greatly from the bulk dataset, especially at the phylum level (Supplementary Figure S2). As expected from the differential abundance procedure, OTUs that are abundant in soil and groundwater do not appear in the set of core-enriched OTUs (Supplementary Table S5).

However, the same taxonomic classification may be represented by distinct OTUs in multiple sample types. For example, a single OTU classified as family Comamonadaceae and 100% identical to *Serpentinimonas maccroryi* (Suzuki et al., 2014; Bird et al., 2021) represents 86–88% of all sequencing reads in the CSW groundwater samples, consistent with previous work at this site (Twing et al., 2017). This OTU is detected at low levels in core samples but is not included in the final set of core-enriched OTUs. Instead, family Comamonadaceae is represented in the core-enriched set by other OTUs that are significantly more abundant in core samples than in groundwater. There are four core-enriched Comamonadaceae OTUs (OTU00003-B, OTU00010-B, OTU00016-B, and OTU00058-B), and the closest relatives of these, assessed as best hits in the NCBI nr database via BLAST analysis, belong to the genera *Acidovorax*, *Diaphorobacter*, and *Variovorax* (Supplementary Table S6). These core-enriched *Comamonadaceae* OTUs share only 93–96% *16S rRNA* gene sequence similarity with the fluid-enriched *Serpentinomonas maccroryi* (NCBI accession MW411452).

Other notable core-enriched OTUs include those classified as *Nitrosomonadaceae* and iron-oxidizing *Gallionellaceae*, which are most abundant in the shallowest core sample (CSW1R; 2.4 mbs) and fall within the *Gammaproteobacteria* bar in Figure 3. Core sample CSW2R (4.2 mbs) had the most diverse array of core-enriched phyla, including Acidobacteria, *Methylomicrobia*, *Myxococcota*, *Verrucomicrobia*, and *Gammaproteobacteria* (Figure 3), the latter of which was composed of the sulfur-oxidizing *Sulfurifustis* (Kojima et al., 2015) and the uncultivated group TRA3-20 (Kim et al., 2021). The deeper CSW cores, CSW10R (19.1 mbs) and CSW14R (25.5 mbs), contained much higher relative abundances of two particular OTUs, the previously mentioned *Acidovorax* OTU (OTU0003-B) and the other classified as *Dechloromonas* (Achenbach et al., 2001).

The same core-enriched *Comamonadaceae* OTU (OTU0003-B) was also abundant in samples of the QV core, but the *Dechloromonas* OTU that is abundant in CSW core samples was absent in QV core samples. The most abundant core-enriched OTU in QV core samples was OTU00019-B, which was classified as *Mycobacteriaceae* and had best hits in the NCBI nr database to *Mycolicibacterium* sp. from a limestone cave (Niyomvong et al., 2012) and hydrocarbon-rich samples (Yu et al., 2015). This single OTU accounts for the majority of Actinobacteria in QV core samples and was also present at low levels in CSW core samples (0.01–0.4% of the total community; Supplementary Table S6). Additionally, the deepest QV core sample (QV42R, 43.3 mbs) also contained two abundant *Burkholderiaceae* OTUs. The first, OTU00234-B, had multiple best hit results belonging to the genus *Polynucleobacter*, which is typically found in freshwater lakes and streams (Hahn et al., 2017; Hahn et al., 2022). The other was OTU00494-B, classified only to the family level, but with best hit results from hydrocarbon-rich samples (Ros et al., 2014; Salam et al., 2018) and rocks (Elser et al., 2015). Neither of these groups were found in other core or groundwater samples, but were present at low levels in soil samples.

### Core-enriched archaea

**Figure 4:**
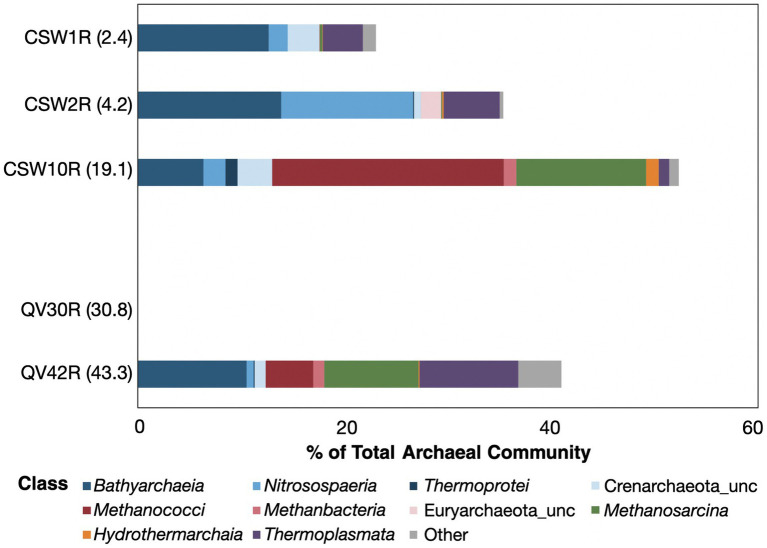
Relative abundance of core-enriched archaeal taxa. The sizes of the bars represent the percent of the archaeal community composed of core-enriched taxa, with white space beyond the bar representing the portion of the archaeal community that could not be attributed to core-enriched taxa (as defined by the differential abundance metrics). The core-enriched taxa are identified at the class level. Archaea were only able to be sequenced from five core samples. All the archaeal taxa identified in QV30R were identical to soil-enriched taxa; therefore, there are no core-enriched taxa in this sample to be represented here.

There were a total of 282 archaeal core-enriched OTUs identified across the dataset (Supplementary Table S5), but only 36 of those were >1% in any given core sample (Supplementary Table S6). The percentage of archaeal OTUs identified as core-enriched ranged from 23–52% in the four core samples for which archaeal sequencing was successful (Figure 4). This excludes core sample QV30R because most of its archaeal OTUs were traced to soil (Figure 2D), and only 1% were identified as core-enriched. The core-enriched archaea of the two shallowest core samples, CSW1R (2.3 mbs) and CSW2R (4.2 mbs), belonged to the *Bathyarchaeia*, *Nitrososphaeria*, *Thermoplasmata*, and unclassified members of the Euryarchaeota (Figure 4). While CSW10R (19.1 mbs) also contained these taxa, it also had much higher relative abundances of *Methanococci*, *Methanobacteria*, *Methanosarcinia*, and *Hydrothermarchaeia*, all groups known to contain methane-cycling archaea. CSW10R was dominated by *Methanoferividicoccus*, a single OTU which comprised 15% of the sample, and three additional OTUs all belonging to the same species comprised another 7% cumulative relative abundance. Similarly, the deepest core in the study, QV42R (43.3 mbs) contained *Bathyarchaeia*, *Nitrososphaeria*, *Thermoplasmata*, *Methanococci*, and *Methanosarcinia*. Additional rare taxa in QV42R (classified as “other” in Figure 4) included *Aenigmarchaeia*, *Lokiarchaeia*, *Archaeoglobi*, *Methanocellia*, *Nanoarchaeia*, and unclassified archaea.

Earlier studies assessing the microbial diversity of serpentine rocks detected only archaea belonging to the *Nitrososphaeria* and *Thermoplasmata* (Supplementary Table S6; Motamedi et al., 2020; Goordial et al., 2021; Daae et al., 2013; Khilyas et al., 2019). Recently, potential methanogens classified as *Methanobacteria* and *Methanosarcina* were reported in serpentinite cores from the Oman Drilling Project (Supplementary Table S6; Kraus, 2021), and highly reducing, high-pH fluids from boreholes established by the Oman Drilling Project contain *Methanobacteria* (Kraus et al., 2021; Thieringer et al., 2023). *Methanococci*, which make up 22% of CSW10R, and *Bathyarchaeia*, which are discussed in detail below, were not detected in any previous studies of serpentinized rocks (Supplementary Table S6).

Abundant core-enriched *Bathyarchaeia* OTUs were found in all four archaea-bearing core samples, ranging from 6–13% of the samples’ archaeal sequencing reads (Figure 4). A total of 102 *Bathyarchaeia* OTUs were “core-enriched” (Supplementary Table S5), and of those, 13 OTUs had relative abundances as high as 1–3% in a given core sample (Supplementary Table S6).

We constructed a phylogeny with these core-enriched *Bathyarchaeia* OTU sequences and reference sequences obtained from public databases. Most of the core-enriched *Bathyarchaeia* OTUs identified here were assigned to subgroups 5a, 6, or 12 (Supplementary Figure S5). These clades have been found in a variety of environments around the world, where they are expected to thrive in variable environmental conditions with metabolic flexibility (Zhou et al., 2018; Fillol et al., 2016; Wang et al., 2020; Hou et al., 2023; Yin et al., 2022; Devereux et al., 2015). Subgroup 6 is a large and diverse clade known for its variety of metabolic pathways, including the ability to synthesize vitamin B_12_ (Hou et al., 2023). This unique capability suggests subgroup-6 may act as an ecological keystone species by supplying this essential cofactor to the surrounding members of their microbial communities (Fillol et al., 2016). Given the limited hydrological circulation in the cores compared to groundwater samples, *Bathyarchaeia* subgroup-6 may play a critical role in sustaining endolithic, nutrient-limited microbial ecosystems by locally producing and supplying vitamin B12.

One OTU from this study was assigned to subgroup 17, which has been found previously in sulfide-rich and reducing environments (Hou et al., 2023; Anantharaman et al., 2016). Metagenome-assembled genomes assigned to subgroup 17 include genes associated with the reductive glycine pathway (Hou et al., 2023) and carbohydrate degradation via the Embden-Meyerhof glycolysis pathway (Lazar et al., 2016). Genes for nitrite reduction (*nirB* and *nirD*) indicate *Bathyarchaeia* subgroup-17’s potential involvement in nitrogen cycling via dissimilatory nitrite reduction to ammonia (Lazar et al., 2016).

Another *Bathyarchaeia* OTU was assigned to subgroup 15, which has been linked to protein degradation in marine sediments (Yin et al., 2022). In addition, key genes for the reductive glycine pathway are also found in this subgroup (Hou et al., 2023), suggesting a capacity for carbon fixation and metabolic flexibility in nutrient-limited environments.

In summary, these results suggest that *Bathyarchaeia* and methanogens could be mediating carbon fixation within subsurface serpentinites, although this hypothesis remains to be tested with metagenomic and metabolic studies. The presence of potentially autotrophic archaea is a new finding for serpentinites and is consistent with similar studies in other geologic settings, where multiple taxonomic groups of archaea attached to subsurface rocks appear to be autotrophic (Lazar et al., 2017).

### Habitats of best hits to core-enriched OTUs

**Figure 5:**
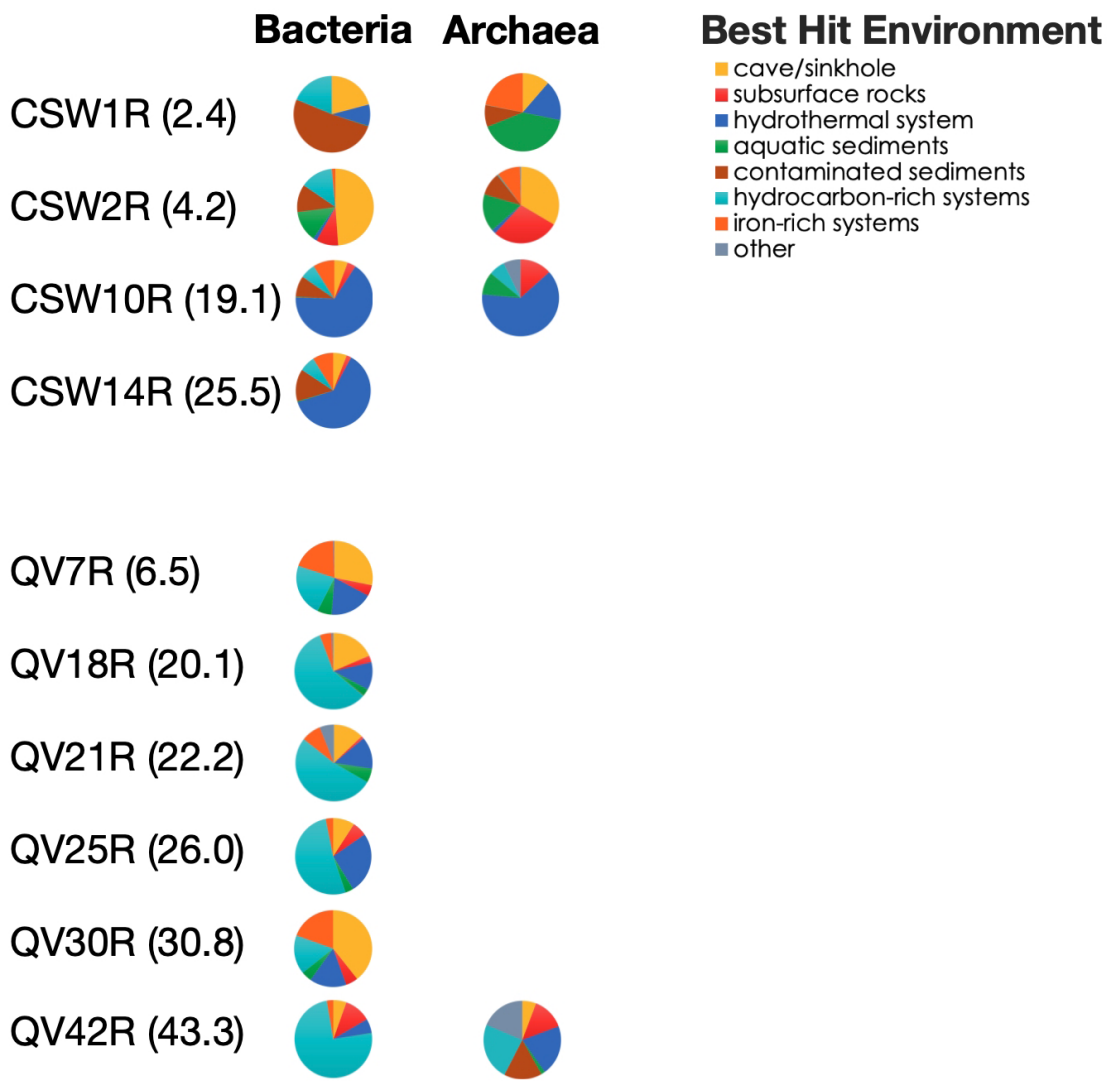
Environment of best hit to core-enriched sequences. Representative sequences from abundant core-enriched OTUs (>1% in any sample) were BLASTed to identify the best hit. The environmental origin of each best hit was identified from the literature and classified into an environmental category. The cumulative relative abundances of core-enriched OTUs are plotted according to the environmental category of their best hit. Note this is only the percentage of the core-enriched OTUs, which constitute a subset of the whole community.

To investigate where the closest matches to the core-enriched OTUs have been found in other studies, we looked up the habitat of the best BLAST hit to each core-enriched OTU. This analysis demonstrated a clear difference between deep rock core samples (19–43 mbs) and shallower samples (2–4 mbs). The best hits of the most abundant core-enriched OTUs in deeper samples were much more likely to be from seafloor hydrothermal vents (deep CSW core samples) and hydrocarbon-rich systems (deep QV core samples) (Figure 5). Meanwhile, the sequences from the shallower samples had best hits that were found in environments such as caves, sinkholes, and sediments from aquatic and contaminated environments (Figure 5).

### Correlations between core-enriched OTUs and mineralogy

The compositions of core-enriched bacterial (but not archaeal) OTUs were significantly correlated with the presence of quartz, albite, and phyllosilicate in core samples (Supplementary Table S7). No significant correlations were evident between the distribution of core-enriched OTUs and the presence of serpentine. Quartz and albite were characteristic of the shallowest CSW core samples, while phyllosilicates were associated with deeper samples. The presence of quartz and albite in both the soils and in some regions of the deeper core at QV may reflect the involvement of non-serpentine rocks, impacted by hydrothermalism. These silica-enriched minerals may represent a relatively more permissive environment, with moderate pH and relatively abundant organic carbon and nitrogen. Alternatively, the interface between the silica-enriched layer and the serpentinites may represent a zone of relatively high hydrological connectivity—a supply of energy and nutrients that is more readily available than the surrounding material. In contrast, the phyllosilicate (clay) minerals in the core material likely represent low permeability regions with little connectivity, and thus a relatively harsh region for microbial activity.

## Conclusion

The results of this study demonstrate that subsurface serpentinite rocks in a continental setting host archaeal and bacterial species that are distinct from those in groundwater and overlying soil. Although the levels of biomass and organic carbon were very low in rock cores, distinct archaeal and bacterial taxa associated with subsurface serpentinites were identified. Notable rock-associated taxa include *Bathyarchaeia*, *Methanococci*, *Methanobacteria*, *Methanosarcinia*, *Sulfurifustis*, and *Dechloromonas*, all of which are expected to be anaerobic and potentially utilizing reduced inorganic compounds (e.g., hydrogen and hydrogen sulfide) that are expected to be abundant in a subsurface, serpentinite-hosted environment. The subsurface rock-associated taxa were identified with a multifaceted statistical approach designed to control for the high risk of contamination in low-biomass samples while acknowledging the inherent heterogeneity and natural mixing of habitat types that occurs in a dynamic environmental system. Future studies should experimentally test the metabolic capabilities of these putative inhabitants of serpentinites and their unique adaptations for life in a high-pH and highly reducing subsurface habitat.

## Data Availability

The datasets presented in this study can be found in online repositories. The names of the repository/repositories and accession number(s) can be found at: https://www.ncbi.nlm.nih.gov/, PRJNA289273, PRJNA1097798.
