# Supplementary material for: Heterogeneity of rock-hosted microbial communities in a serpentinizing aquifer of the Coast Range Ophiolite
Source: Front Microbiol. 2025 Mar 7;16:1504241. doi: 10.3389/fmicb.2025.1504241 (PMC11926711; doi:10.3389/fmicb.2025.1504241)
Supplement: Supplementary file 8 [file Data_Sheet_1.DOCX]

**
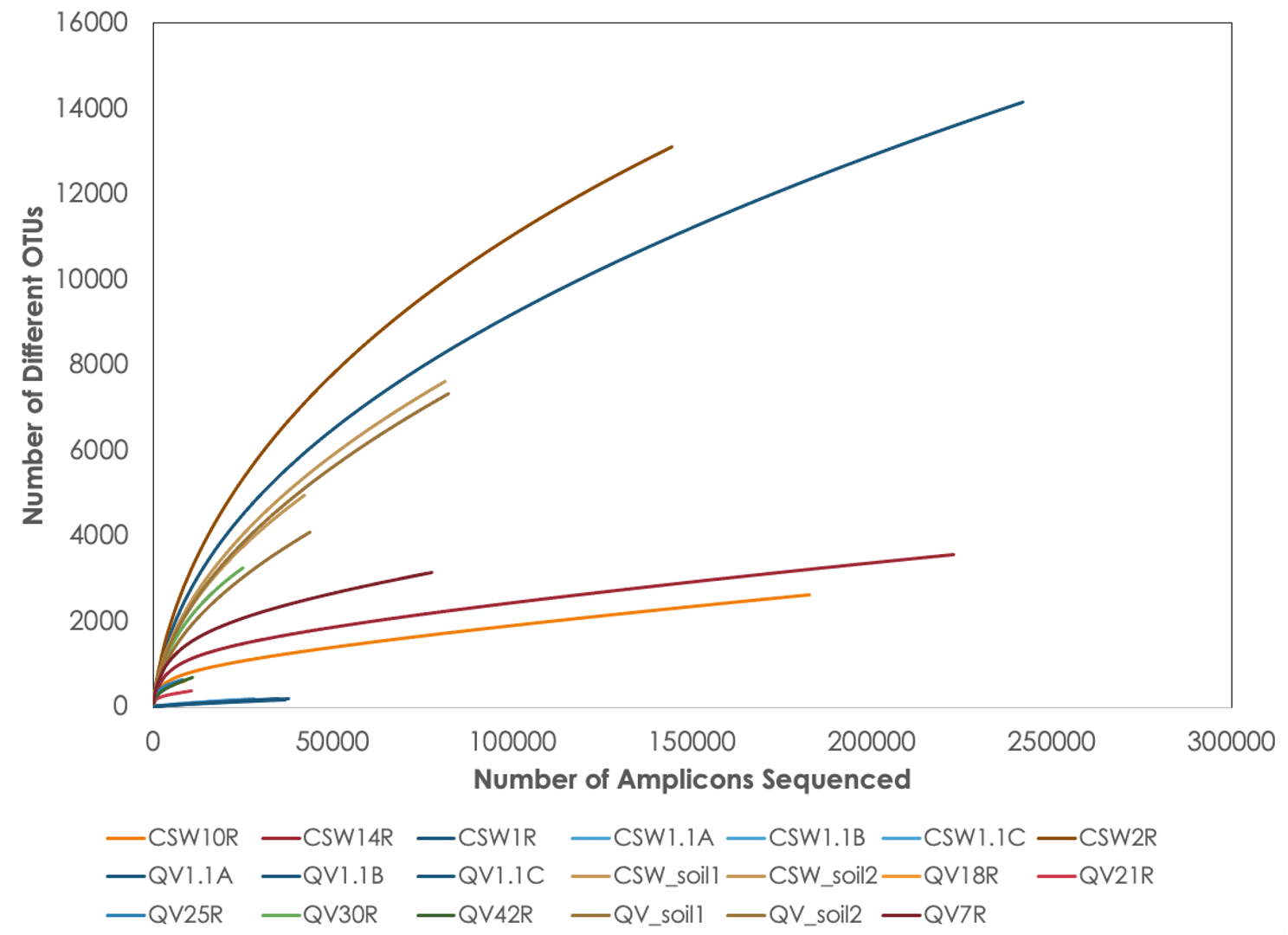
**


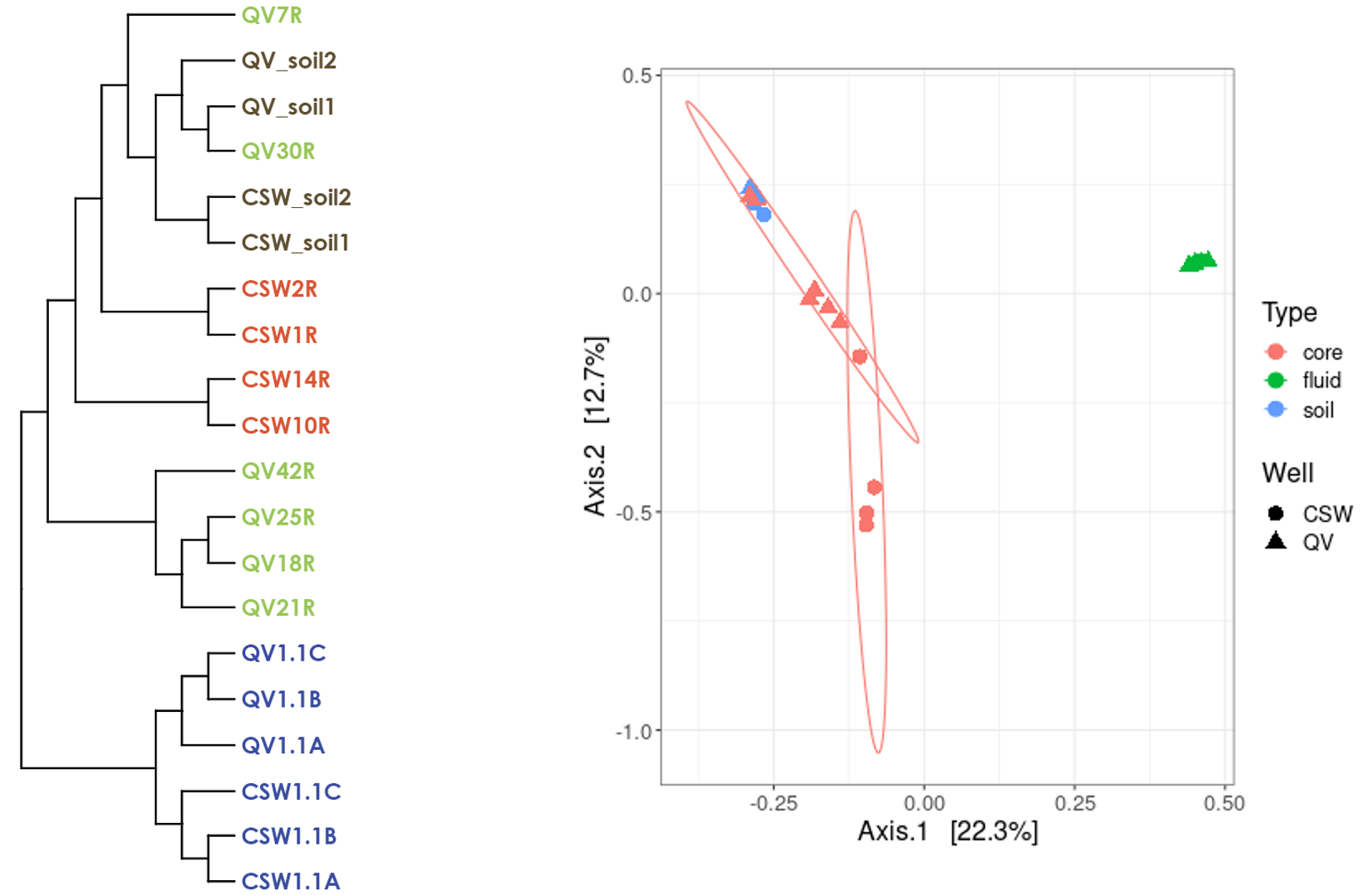


##### **Figure S1 - Alpha- and beta-diversity measures of bacterial dataset.**

(A) Rarefaction analysis of bacterial 16S rRNA gene amplicon sequences. (B) Community similarity dendrogram calculated from Sørenson diversity index. (C) MDS plot of bacterial community similarity calculated from the Bray-Curtis diversity index.

#####
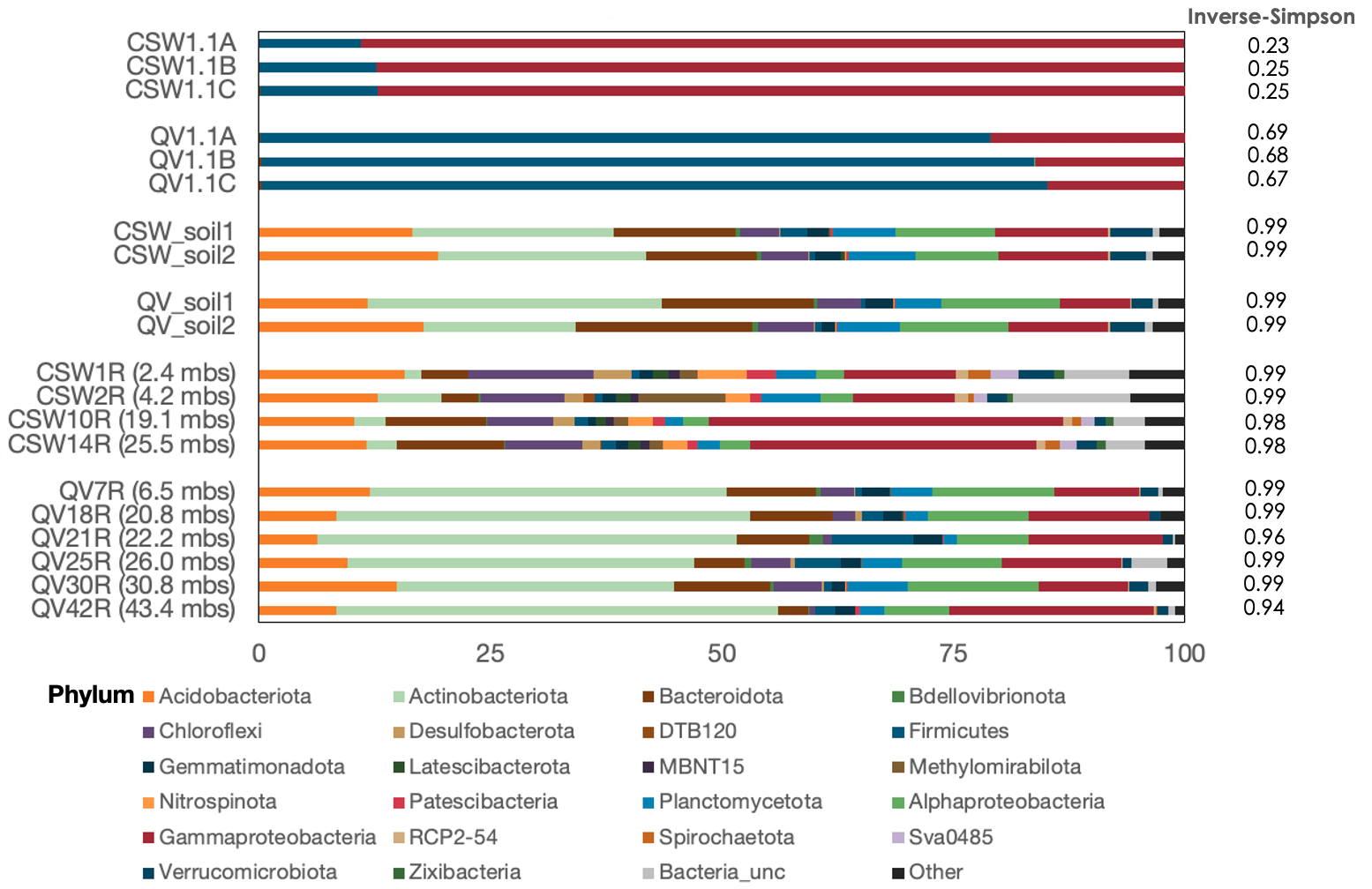
**Figure S2 - Relative abundance of bacteria in the fluid, soil, and core samples at the Phylum level.**

Due to its disproportionate abundance, the phylum Proteobacteria is represented at the Class level with *Alphaproteobacteria* and *Gammaproteobacteria*. No other classes of the Proteobacteria were detected. ‘Other’ represents phyla which were not more than 1% relative abundance of a single sample. Depths of core samples are included in parentheses beside the sample name.

##

#####
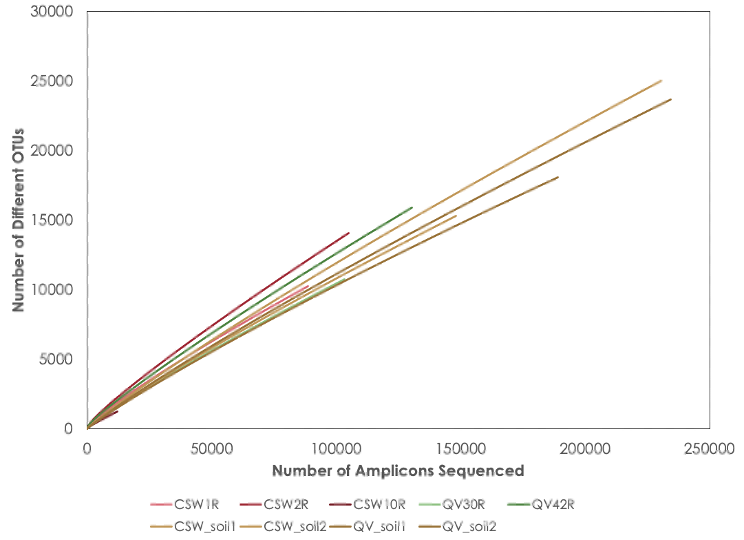
**
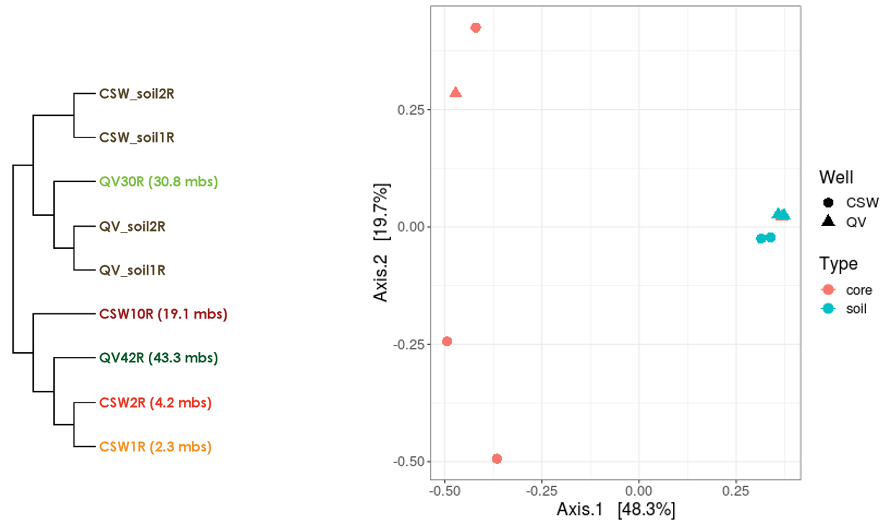
Figure S3 - Alpha- and beta-diversity measures of archaeal dataset.**

(A) Rarefaction analysis of archaeal 16S rRNA amplicon sequences. (B) Community similarity dendrogram calculated from Sørenson diversity index. (C) MDS plot of archaeal community similarity calculated from the Bray-Curtis diversity index.

## **
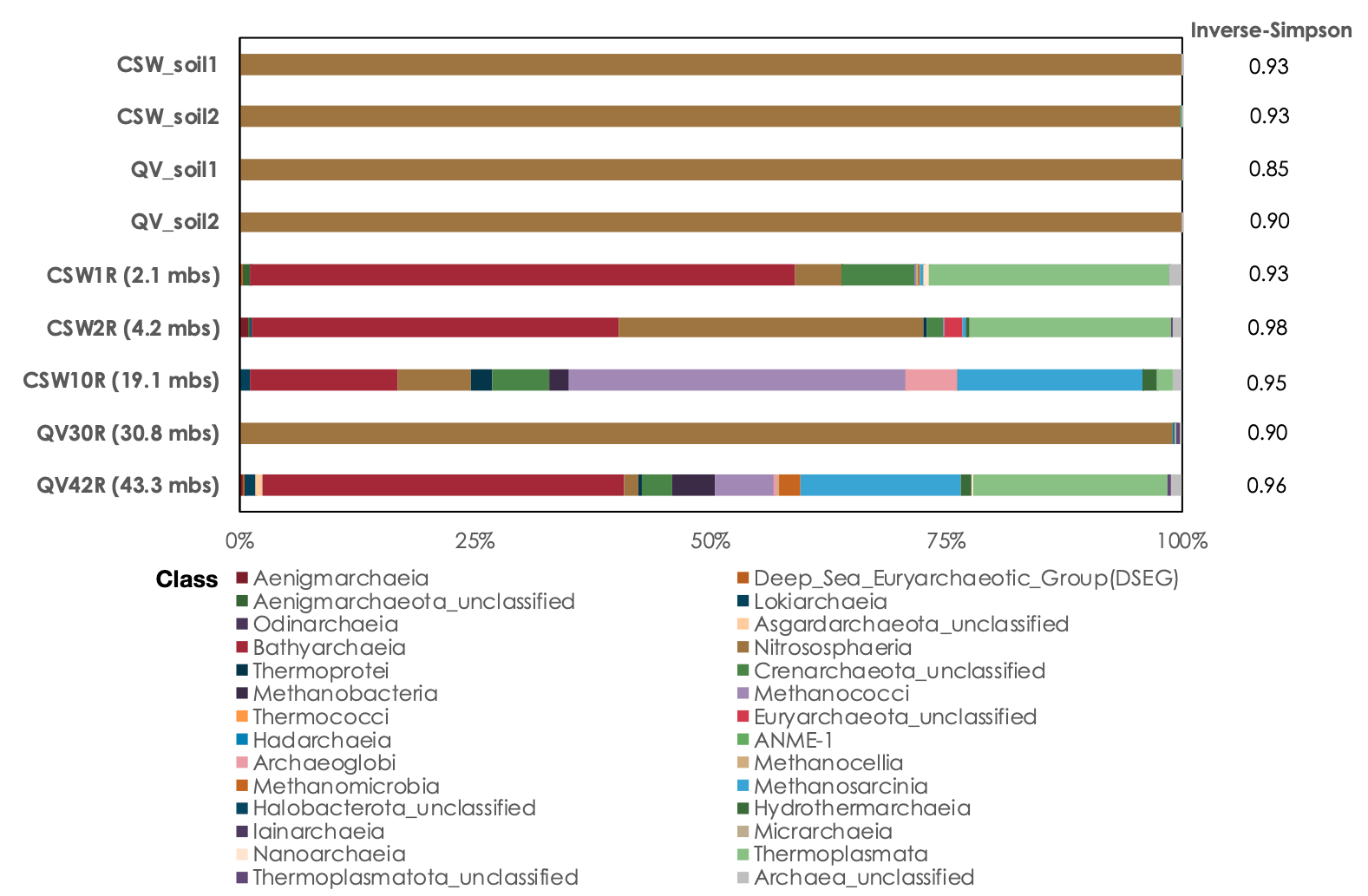
**

##### **Figure S4 - Relative abundance of archaea at the Class level.** Depths of core samples are included in parentheses beside the sample name.

##### **
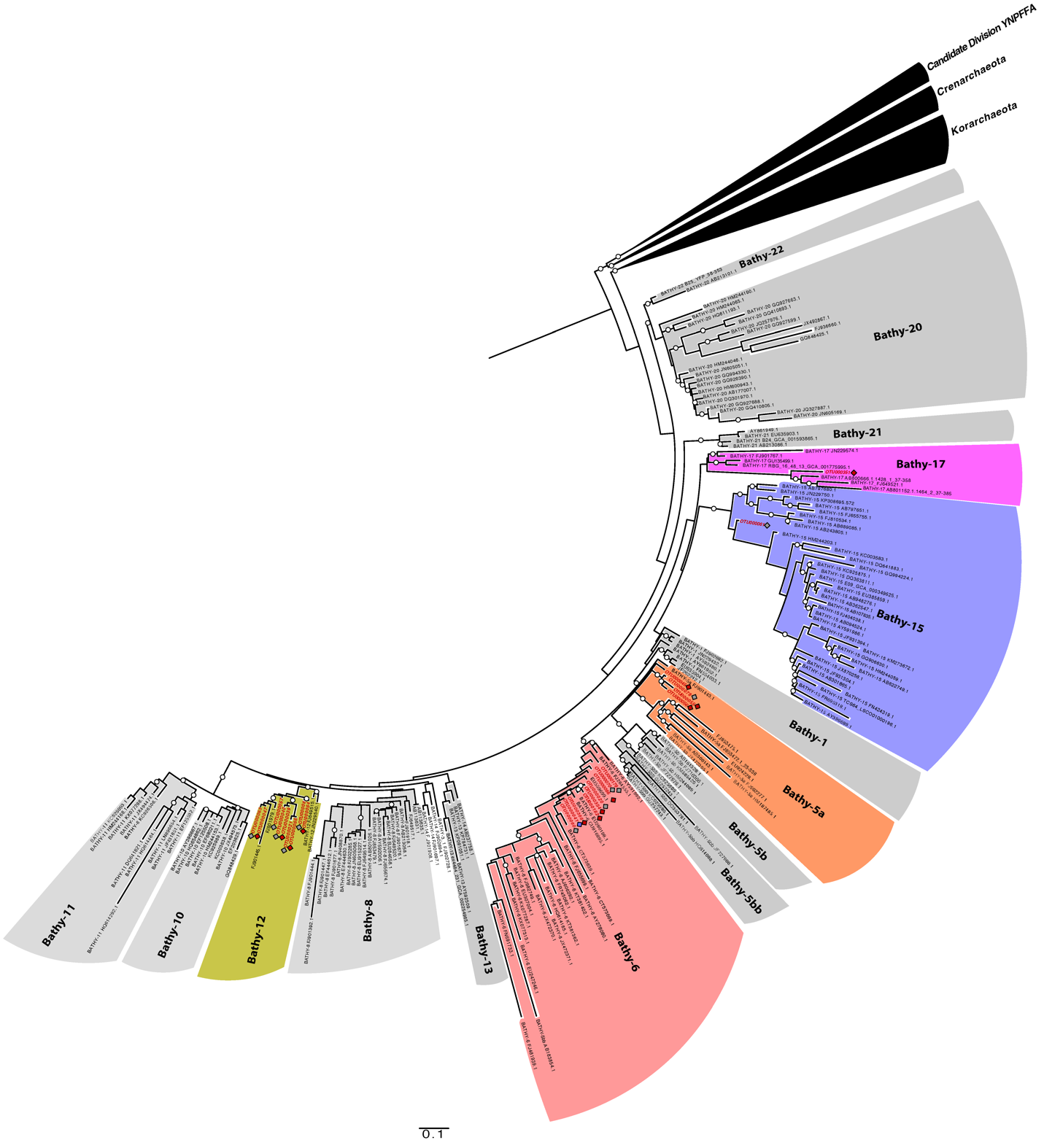
****Figure S5 - 16S rRNA phylogenetic tree of Bathyarchaeota OTUs.** OTUs from this study are highlighted in red and represented in non-gray Bathyarchaeota subgroups. Based on concatenated alignment of 16S rRNA gene V4-V6 hypervariable regions of representative sequences from this study and Zhou et al. 2018. *Crenarchaeota*, Korarchaeota, and Candidatus YNPFFA sequences are referenced as outgroups. Bootstrap values > 0.7 are shown with white dots. Core-enriched OTUs exclusive to CSW and QV30 cores are indicated by red and blue diamonds, respectively. OTUs marked by gray diamonds are found both in cores.

**References**

Zhou Z, Pan J, Wang F, Gu JD, Li M. Bathyarchaeota: globally distributed metabolic generalists in anoxic environments. FEMS Microbiology Reviews. 2018 Sep 1;42(5):639–55.
